# Letters to the Journal

**Published:** 1903-11

**Authors:** 


### Letters to the Journal.

Here is a very personal letter. Its publication (with permission of the writer, of course) may be criticized as a species of vanity on my part. Well, let it. Such sincere and outspoken appreciation of one's labors in a good cause is rare, and when it comes it is as balm and strong wine. It is hard to resist the temptation to let certain of the doctors in Texas see this letter—especially those who accept the JOURNAL as long as I will send it, but who refuse to pay for it. The love of approbation is inborn in us; nay, even in the dog. If your dog *does his best,* he expects a pat, and is disgusted if he receives a kick instead. Such letters are always gratifying; but, coming as it does from a distinguished physician, who is universally esteemed as a type of the cultivated gentlemen of the South and an ethical physician, who exemplifies in his life the highest ideal of a true disciple of the healing art, this letter is doubly gratifying; it comes like a benediction, and stimulates to renewed and continued efforts in behalf of legitimate medicine, the public health, and a pure and high standard of professional life, things to which the JOURNAL has devoted nearly a quarter of a century of its most earnest advocacy and effort.

DALLAS, TEXAS, October 9, 1903.

*Dear Doctor Daniel:*

Please find enclosed $2.00, subscription for the "Red-Back" for the current year. I know your price for the JOURNAL is only $1.00, and, if you have any scruples about accepting the amount enclosed, please place the extra dollar to the credit of some unfortunate delinquent. Your JOURNAL is, in my opinion, one of the very best of the time—fully up to the standard and abreast with the progress and advancement of the day. You have managed to secure some very able contributors, and your selections have been very judicious and practical. Your own contributions have been of the very highest order—most instructive and valuable—always dictated in the most ornate style, thus rendering them attractive and fascinating. It has been a matter of wonderment to me how, with the very moderate means at your command, you could get up such a perfect little gem in its way—now the pride and admiration of the profession of Texas. Not only this, my dear Doctor, but you have done more, by pen and tongue and worthy example, to refine, ennoble and elevate the profession of our State than any score of its members. Surely you are entitled to our highest esteem, gratitude and honor. These expressions of appreciation come from the bottom of my heart, and it is a pleasure to communicate them to one so worthy.

Best wishes and kindest regards for you and yours, my dear Doctor.

Very truly, your friend,

S. EAGON.

